# Experimental data on the properties of natural fiber particle reinforced polymer composite material

**DOI:** 10.1016/j.dib.2017.06.020

**Published:** 2017-06-17

**Authors:** D. Chandramohan, A. John Presin Kumar

**Affiliations:** Department of Mechanical Engineering, Hindustan Institute of Technology & Science, Chennai, Tamil Nadu 603103, India

**Keywords:** Hybrid, Polymer matrix composites (PMCs), Mechanical properties, Mechanical testing, Coconut shell powder, Walnut shells powder, Rice husk powder

## Abstract

This paper presents an experimental study on the development of polymer bio-composites. The powdered coconut shell, walnut shells and Rice husk are used as reinforcements with bio epoxy resin to form hybrid composite specimens. The fiber compositions in each specimen are 1:1 while the resin and hardener composition 10:1 respectively. The fabricated composites were tested as per ASTM standards to evaluate mechanical properties such as tensile strength, flexural strength, shear strength and impact strength are evaluated in both with moisture and without moisture. The result of test shows that hybrid composite has far better properties than single fibre glass reinforced composite under mechanical loads. However it is found that the incorporation of walnut shell and coconut shell fibre can improve the properties.

**Specifications Table**

**Table:** 

Subject area	*Materials science*
More specific subject area	*Natural fibre composites*
Type of data	*Text file, tables, graphs and figures*
How data was acquired	*Mainly by a series of experimental (laboratory) investigations*
Data format	*Raw, calculated, analyzed, tabulated, plotted*
Experimental factors	*Mechanical and fracture properties composite material*
Experimental features	*In this all composites specimen were under goes flexural, tensile, impact and hardness test in both wet and dry condition*
Data source location	*School of Mechanical Sciences,Hindustan University,Chennai,Tamilnadu,India*
Data accessibility	*Data is with the article.*

**Value of the data**

• This data set presents a complete mechanical characterization of a hybrid composites.
• Guidelines for the mechanical and fracture characterization of a hybrid composites material are provided.
• Mechanical properties of composite material were determined in both with moisture and without moisture condition

## 1 Data

Authors׳ presented all data in following tables and figure׳s by experiments.

## 2 Experimental design, materials and methods

### 2.1 Materials

The matrix material used in this investigation was bio epoxy resin Grade 3554A and Hardner 3554B. Supplied by Lab chemicals, Chennai and reinforcements were obtained from nearby local market as raw form as shown in Table 1. The said fibres were cleaned by water then they are powdered by grinding.

**Table 1:** Agricultural waste material.

### 2.2 Methods

The said fibres (Table 2) were cleaned by water then breaking the shells into pieces and they were first ground in a ball mill to produce fibre powder and then separated by mechanical sieving into particle form.

**Table 2:** Materials.

**Material**	**Type**	**Supplied by**
Matrix	Bio Epoxy resin	Lab chemicals, Chennai
Catalyst	Hardner	
Releasing agent	Poly vinyl acetate	
Coconut shell, walnut shell and Rice husk	Particle	The Coconut shell, walnut shells & Rice husk were obtained from nearby local market.

#### 2.2.1 Chemical treatment of said natural fibres

The fibres are powdered. Then the fibres are cleaned normally in clean running water and dried. A glass beaker is taken and 1% NaOH is added and 99% of distilled water is added and a solution is made. After adequate drying of the fibres in normal shading for 2 to 3 h, the fibres are taken and soaked in the prepared NaOH solution [1]. Soaking is carried out for different time intervals depending upon the strength of fibre required. In this study, the fibres are soaked in the solution for three hours. After the fibres are taken out and washed in running water, these are dried for another 2 h. The fibres are then taken for the next fabrication process namely the Procasting process.

#### 2.2.2 Advantages of chemical treatment

Chemical treatment with NaOH removes moisture content from the fibres thereby increasing its strength. Also, chemical treatment enhances the flexural rigidity of the fibres. Last, this treatment clears all the impurities that are adjoining the fibre material and also stabilizes the molecular orientation.

#### 2.2.3 Preparation of specimens

A mould is made of wood in dimensions given in Table 3 ASTM Standards for Specimen Preparation. An OHP sheet is taken and releasing agent is applied over it and fitted with the inner side of the mould and allowed it to dry. A glass beaker and a glass rod or a stirrer are taken and cleaned well with running water and then with warm water. Thereafter the calculated quantity of bioepoxy resin and the measured quantity of hardener (ratio 10:1) are added to a breaker and the mixture is stirred for nearly 25 min. The reason behind this stirring is to create a homogeneous mixture. Then, after the mixing is over, the calculated quantity of fibers (ratio 1:1) is added and stirring process is continued for the next 45 min. Then the mixture is poured into the mould and rammed mildly for uniform settlement. Then the mould is allowed to solidify for nearly 24 h.

**Table 3:** Standards for specimen preparation.

**Test**	**Specimen standards**	**Schematic figure as per ASTM standard**
Tensile strength	ASTM D638	
Flexural strength	ASTM D790	
Impact Test	ASTM D256	
Double shear test	ASTM D5379	

#### 2.2.4 Water absorption test

The effect of water absorption is important incase the material that has been developed when used for applications comes in contact of water. The composite specimens to be used for moisture absorption test were first dried in an air oven at 50 °C. Then these conditioned composite specimens were immersed in distilled water at 30 °C for about 5 days. At regular intervals, the specimens were removed from water and wiped with filter paper to remove surface water and weighed with digital balance of 0.01 mg resolution. The samples were immersed in water to permit the continuation of sorption until saturation limit was reached. The weighing was done within 30 s in order to avoid the error due to evaporation. The test was carried out according to ASTM D570 to find out the swelling of specimen [2]. After 5 days, the test specimens were again taken out of the water bath and weighed. The weight gain of the sample was measured as increase in weight % using the formula.

Increaseinweight%=(wetweight-sampleweight)/sampleweigh×100

**Fig. 1:**
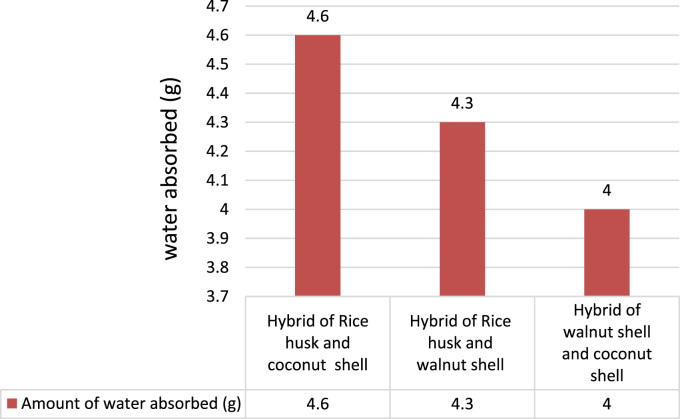
Water absorption results.

Water absorption capacity is another crucial factor to be taken into account when considering the effect of water on the composite material developed. The effect of water absorption is important incase the material that has been developed when used for applications comes in contact of water. From the observation (Fig. 1) it can be concluded that the hybrid of walnut shell and coconut shell composite can absorb less moisture (4 g) for different application environment.

#### 2.2.5 Tensile strength

**Fig. 2:**
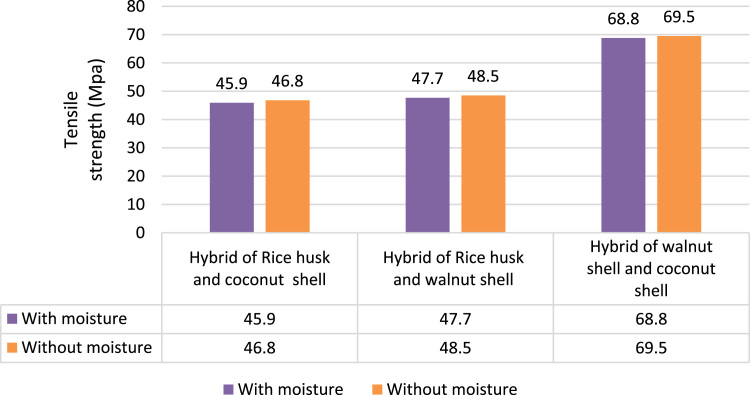
Comparison of tensile strength between different hybrid composites.

The tensile test carried out as per ASTM D638 using Universal Testing Machine, 5569A, Instron. The speed to test was 5 mm/min [3]. The tensile strength of hybrid of walnut shell and coconut shell composite is the relatively more than other two hybrid composites. In both with and without moisture it has a value of 68.8 KN and 69.5 KN respectively as shown in Fig. 2.

#### 2.2.6 Flexural strength

**Fig. 3:**
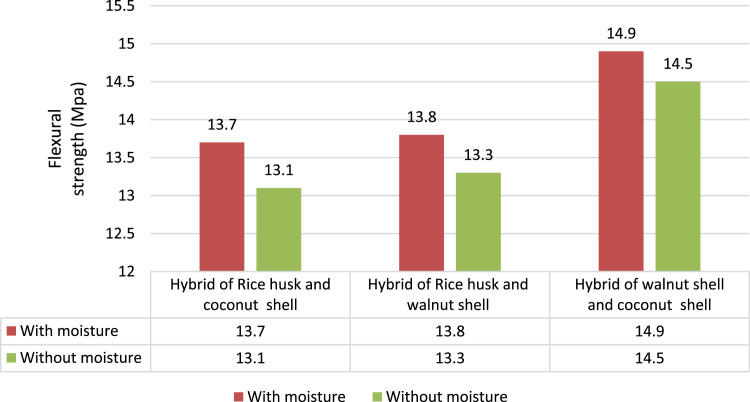
Comparison of flexural strength between different hybrid composites.

The flexural test carried out as per ASTM D790 using Universal Testing Machine, 5569A, Instron. The span length was 50 mm and speed to test 2 mm/min [4]. In both with and without moisture test condition the Hybrid of walnut shell and coconut shell composite has the highest flexural strength (14.9 MPa in wet and 14.5 MPa in dry condition) since its strength increases with increase in interfacial adhesion as shown in Fig. 3 compared to other two hybrid composites.

#### 2.2.7 Impact test

**Fig. 4:**
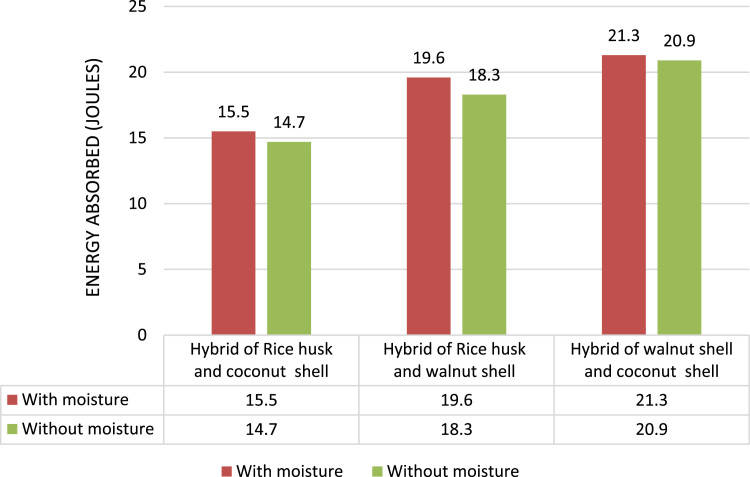
Comparison of Impact test results between different hybrid composites.

The impact test carried out as per ASTM D256 using Impactor II, Ceast. The 5.5 J pendulum was used for the test [4]. In both with and without moisture test condition the Impact strength of Hybrid of rice husk and coconut shell (absorbed 15.5 J wet and 14.7 J in dry condition) and Hybrid of rice husk and walnut shell composites (absorbed 19.6 J in wet and 18.3 J in dry condition) in impact testing is found to be less than that of the Hybrid of walnut shell and coconut shell composite (absorbed 21.3 J in wet and 20.9 J in dry condition) as shown in Fig. 4.

#### 2.2.8 Shear strength

**Fig. 5:**
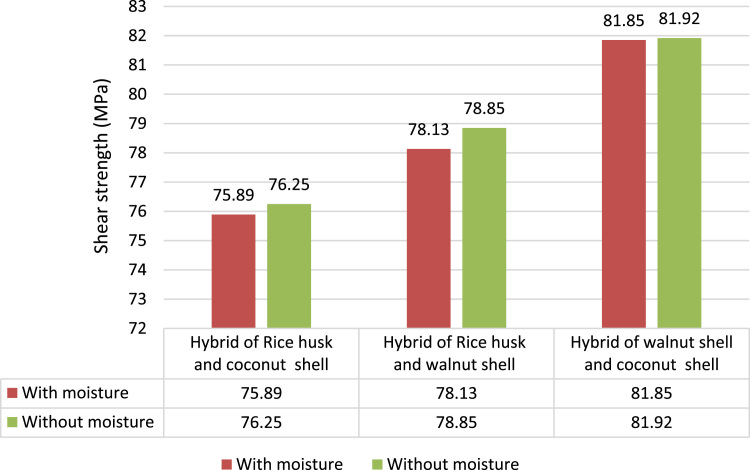
Comparison of shear strength between different hybrid composites.

Double shear test is done as per ASTM standard (ASTM: D5379). Universal testing machine is used for performing shear test with a special fixture for double shear testing [4]. The specimen is held between the grippers and test is performed. In both with and without moisture test condition the shear strength of Hybrid of rice husk and coconut shell (75.89 Mpa wet and 76.25 MPa in dry condition) and Hybrid of rice husk and walnut shell composites (78.13 Mpa wet and 78.25 MPa in dry condition) in shear testing is found to be less than that of the Hybrid of walnut shell and coconut shell composite (81.85 Mpa wet and 81.92 MPa in dry condition) as shown in Fig. 5.

#### 2.2.9 % of elongation of different hybrid composites

**Fig. 6:**
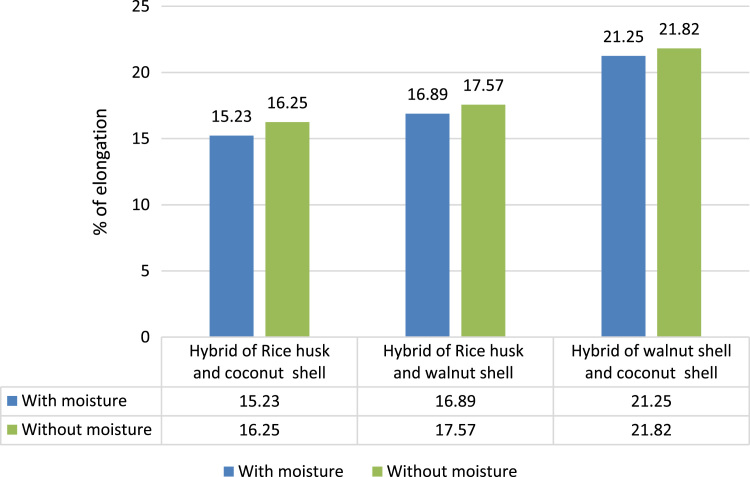
Comparison of % of elongation between different hybrid composites.

Fig. 6 shows the percentage elongation of Hybrid of rice husk and coconut shell and Hybrid of rice husk and walnut shell composites in tensile testing is found to be less than that of the Hybrid of walnut shell and coconut shell composite (21.25% in wet and 21.82% in dry condition). Therefore, Hybrid of walnut shell and coconut shell composite withstands more strain before failure in tensile testing than the other two hybrid composite.

#### 2.2.10 Comparison between different hybrid composites: break load

**Fig. 7:**
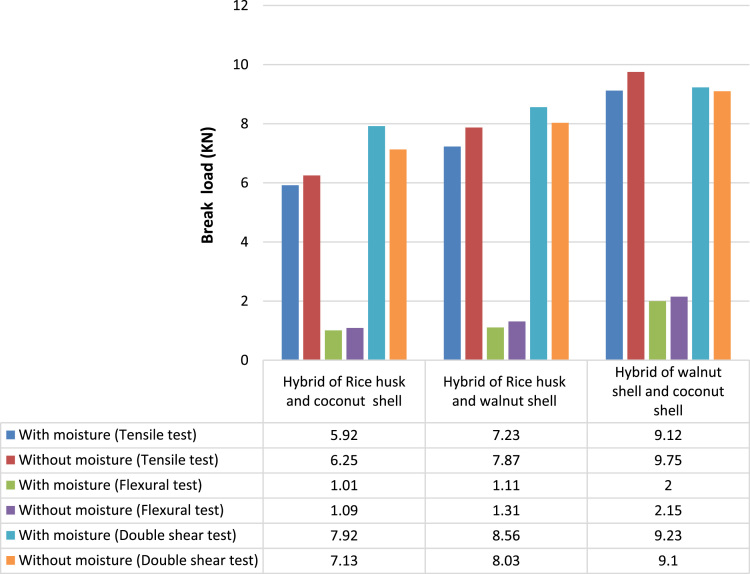
Comparison between different hybrid composites: break load.

The hybrid of walnut shell and coconut shell composite failed at 9.12 kN in tensile test (with moisture), 9.75 kN in tensile test (without moisture) and in flexural test 2 kN and 2.15 kN the said composites failed in with and without moisture respectively. In double shear test also 9.23 kN and 9.1 kN the said composites failed in with and without moisture respectively. From this observation the hybrid of walnut shell and coconut shell composite withstand maximum load compared to other two hybrid composites as shown in Fig. 7.

From the experimental results hybrid of 3 different specimen possess good mechanical properties and there is no significant different in both with and without moisture test results. However it is found that the incorporation of walnut shell and coconut shell fibre can improve the properties. This work can be further extended to study other tribological aspects like abrasion, wear, hardness behavior of this composite. We can also study other aspects of such composites like use of other potential fillers for development of hybrid composites and evaluation of their mechanical and erosion behavior and the resulting experimental findings can be similarly analyzed. Investigation of the powder morphology and fracture surfaces of composite specimens will be carried out by using scanning electron microscopy.

Generating wealth from waste such as from the rice husk, coconut shell and walnut shell should be regarded as one of the ways to create an eco-friendly environment for the future generations [5]. It is vital that each one offered by-products be changed into extremely business outputs so as to sustain this natural resource and supply extra financial gain to small scale farming industries while not compromising its quality and safety in competitive with different business product.
